# Yangxue Jiedu Fang Ameliorates Psoriasis by Regulating Vascular Regression via Survivin/PI3K/Akt Pathway

**DOI:** 10.1155/2021/4678087

**Published:** 2021-01-08

**Authors:** Hongpeng Lv, Xin Liu, Weiwen Chen, Shiju Xiao, Yunrun Ji, Xuyang Han, Yafan Li, Xiaoxu Wang, Guangzhong Zhang, Ping Li

**Affiliations:** ^1^Beijing Hospital of Traditional Chinese Medicine, Capital Medical University, Beijing 100010, China; ^2^Beijing University of Chinese Medicine, Beijing 100029, China; ^3^Beijing Institute of Traditional Chinese Medicine, Beijing 100010, China; ^4^Graduate School, Capital Medical University, Beijing 100069, China

## Abstract

**Background:**

Psoriasis (PA) is a chronic autoimmune disease of the skin that adversely affects patients' quality of life. Yangxue Jiedu Fang (YXJD) has been used for decades to treat psoriasis in China. However, its antipsoriatic mechanisms are still poorly understood. In this study, we explored the effects of YXJD on angiogenesis and apoptosis of microvessels in PA, the underlying mechanisms in HUVEC cells transfected by Survivin overexpression plasmid and in a mouse model of imiquimod-induced psoriasis and the relationship between VEGF (vascular endothelial growth factor) and Survivin.

**Methods:**

A BALB/c mouse model of imiquimod- (IMQ-) induced PA was established, and the mice were treated with YXJD. Cell viability was assessed by CCK8 assay. Apoptosis was detected by annexin V–FITC/PI double-staining and caspase-3 assays. The PI3K/Akt/*β*-catenin pathway was analyzed by western blotting, ELISA, and immunochemical analysis.

**Results:**

YXJD ameliorated symptoms and psoriasis area and severity index (PASI) scores and also reduced the number of microvessels, as determined by the microvessel density (MVD). The expression of apoptotic protein Survivin in endothelial cells, autophagy-related proteins p62, and angiogenic proteins VEGF was inhibited by YXJD, and the repressed expression of LC3II/I increased by YXJD. The proteins related to the PI3K/Akt pathway and *β*-catenin expression and the nuclear entry of *β*-catenin were reduced in IMQ-induced PA mice treated with YXJD. In HUVEC cells transfected by Survivin overexpression plasmid, we observed YXJD regulated the expression of Survivin, LC3II/I, and p62, VEGF, and PI3K/Akt pathway-relative proteins and the nuclear entry of *β*-catenin.

**Conclusions:**

YXJD inhibited the expression of Survivin via PI3K/Akt pathway to adjust apoptosis, autophagy, and angiogenesis of microvessels and thus improve the vascular sustainability in psoriasis. YXJD may represent a new direction of drug research and development for immunomodulatory therapy for psoriasis.

## 1. Introduction

Psoriasis (PA) is a chronic, autoimmune skin disease that is recognized as a major global health problem by the World Health Organization, and it affects 2-4% of the population [1]. PA is characterized by hyperkeratosis, an increased number of microvessels, tortuous morphology, and the infiltration of lymphocytes around the superficial blood vessels of the dermis [2]. Apoptosis/autophagy and angiogenesis in microvessels of the dermis of normal skin are balance. In psoriatic lesions, the disorder in vascular regression, which is the result of microvessels apoptosis, autophagy, and angiogenesis, results in red plaques on the skin.

Our previous study shows several specific plasma miRNA-targeted pathways associated with psoriasis, such as the VEGF, PI3K/Akt, and WNT signaling pathways, which are regulating angiogenesis in psoriasis [3]. We also found the amelioration in angiogenesis restricted the occurrence, persistence, and recurrence of psoriasis. Yangxue Jiedu Fang (YXJD), a Chinese traditional formula used in the clinic to treat PA in China, was previously evaluated by double-blind randomized controlled clinical trials, and its total effective rate was found to be 67.09% [4]. Our previous experimental studies showed that YXJD can reduce the expression of VEGF, VEGFR, and related mRNAs via the ERK/NF-*κ*B pathway and inhibit the proliferation and migration of HDMECs, which plays a therapeutic role in the pathology of angiogenesis in psoriasis [5]. The active ingredients of YXJD can inhibit angiogenesis. The total flavonoids of purified methanol extracts of *Spatholobi caulis* (one of the herbs in YXJD) (PSC) present proangiogenic activity both in zebrafish and HUVECs [6]. Tanshinone IIA (the main active ingredient of *Salvia miltiorrhiza*, which is one of the herbs in YXJD) effectively inhibits the secretion of VEGF and bFGF in HUVECs and a mouse colon tumor model and can suppress proliferation, tube formation, and metastasis in vitro [7].

Overexpressing Survivin tumor cells induce the synthesis and secretion of VEGF as well as microvascular hyperplasia [8]. Survivin is currently the strongest known inhibitor of apoptosis. It can inhibit apoptosis by inhibiting the signaling pathway regulated by the caspase family of apoptotic proteins [9]. The expressions of Survivin, VEGF, and Akt in keratinocytes of psoriatic lesions in humans were significantly upregulated, and the expression of Survivin and Akt gradually elevated with the increase of PASI score. Survivin increased *β*-catenin-Tcf/Lef transcription through the PI3K/Akt pathway and promoted the generation of VEGF, thereby promoting tumor microvessel formation induced by the secretion of VEGF [10], which we call Survivin/PI3K/AKT pathway. However, it is still unclear whether Survivin can interfere with the PI3K/Akt pathway leading to the occurrence and development of psoriasis. Other research shows the herbs in YXJD can also promote apoptosis. *Spatholobi caulis* tannin (SCT) can mediate related circRNAs to inhibit proliferation and promote apoptosis in HeLa cells, as determined by techniques such as bioinformatics analysis of relevant genes [11]. Tanshinone IIA can also inhibit the activity of the PI3K/AKT pathway and the expression of VEGFR, GSK-3*β*, and apoptosis-related proteins such as Bax, Bcl-2, and caspase-3 in vitro [12–14].

In this study, we investigated the effects of YXJD on apoptosis/autophagy and angiogenesis in vivo and vitro and its underlying mechanisms via the Survivin/PI3K/AKT pathway. Mouse model was established by imiquimod (IMQ) and in cell experiment HUVEC cells transfected with Survivin overexpression plasmid.

## 2. Materials and Methods

### 2.1. Animal

Twenty-four specific pathogen-free (SPF) male BALB/c mice (19 to 21 g, 8 week-old) were purchased from the Beijing WTLH Experimental Animal Technology Co., Ltd., (China) (certification No. SCXK (Beijing) 2012-0001). Mice were housed in the SPF conditions with a relative humidity of 60% and at a temperature of 25°C and had free access to food and water. All animal experiments were performed in accordance with the National Institutes of Health Guidelines on Laboratory Research and approved by the Animal Care Committee of Capital Medical University, Beijing, China (approval number: 2019060201).

### 2.2. Preparation of Herb Extract

The preparation of YXJD contains 10 kinds of traditional Chinese herbal medicine, which are *Salvia miltiorrhiza Bge* (15 g; Beijing Xinglin Pharmaceutical Co. Ltd., Beijing, China), *Angelica Sinensis* (15 g; Beijing Xinglin Pharmaceutical Co. Ltd., Beijing, China), *Rehmannia glutinosa Libosch* (15 g; Beijing Xinglin Pharmaceutical Co. Ltd., Beijing, China), *Ophiopogon japonicus* (10 g; Beijing Xinglin Pharmaceutical Co. Ltd., Beijing, China), Scrophularia ningpoensis Hemsl (15 g; Beijing Xinglin Pharmaceutical Co. Ltd., Beijing, China), *Spatholobi Caulis* (15 g; Beijing Xinglin Pharmaceutical Co. Ltd., Beijing, China), *Smilax glabra Roxb*. (15 g; Beijing Xinglin Pharmaceutical Co. Ltd., Beijing, China), *Paris polyphylla* (9 g; Beijing Xinglin Pharmaceutical Co. Ltd., Beijing, China), *Wrightia laevis* (15 g; Beijing Xinglin Pharmaceutical Co. Ltd., Beijing, China), seed of Asiatic plantain (15 g; Beijing Xinglin Pharmaceutical Co. Ltd., Beijing, China). The herbs were decocted with pure water boiled for 1.5 h (1500 mL). Filtered water extract was concentrated to 513 mg/mL under reduced pressure and then reconstituted in distilled water to achieve the required dose for all subsequent experiments.

### 2.3. Fingerprint Analysis of YXJD through Ultraperformance Liquid Chromatography-Tandem Mass Spectrometry (UPLC-MS/MS)

UPLC-MS/MS was used to analyze the chemical composition and the stability of YXJD. Chromatographic analysis was performed (Agilent Technologies, USA) on a XS205 Triple Quadrupole Mass Spectrometer (Mettler Toledo Technologies, USA). The analytes of YXJD were separated on a ACQUITY UPLC BEH C18 column (2.1 mm × 5 mm, 1.8 *μ*m) with a mobile phase consisting of acetonitrile (A) and 0.1% aqueous solution of formic acid (B); the following gradient program was used: 2% A–27% A at 0–8.5 min, 27% A–100% A at 8.5–10 min, 100% A–2% A at 10–13 min. The injection volume was 5 *μ*L, the flow rate is 0.4 mL/min, and the column temperature was 45°C.

### 2.4. Preparation of Psoriasis Mice Models and Drug Administration

The mice were randomly separated into the following four groups (six mice per group) including the control group, psoriasis model group, YXJD group (10 mL/kg), and cyclosporin group (80 mg/kg). Psoriasis model, a model of IMQ-induced psoriasis, was established by administering a daily topical dose of 62.5 mg of a cream preparation containing 5% IMQ (Mingxinlidi Laboratory, China) on the hair-free back of the mice, in which the area size is 3 cm × 4 cm [13]. Mice in the control group were smeared with the same dose of Vaseline (Baotou Kunlun Petrochemical Co., Ltd.) once a day continuously for 6 days and was orally administered daily with 10 mL/kg saline twice a day. Except for the control group, the other three groups were all established the psoriasis model by applying IMQ continuously for 6 days. The psoriasis model group was given saline (10 mL/kg) intragastrically until the detection time point. The YXJD group was orally administered with YXJD twice per day (10 mL/kg); the cyclosporin group was applied cyclosporine A capsule (Solarbio) one time every 3 days on the day of the first cream application. After six days, mice were sacrificed by cervical dislocation under sodium pentobarbital anesthesia, and skin lesions and serum samples were collected. The corresponding skin lesions were cut off in each group, some of them were fixed in 10% formaldehyde solution to prepare paraffin sections, and the other parts were placed in the refrigerator at-80°C.

### 2.5. Evaluation of Lesion Symptoms

Psoriasis Area Severity Index (PASI) as a modified scoring system for monitoring the severity was used in the study. Erythema, scaling, and thickening were scored independently on a scale from 0 to 4: 0, none; 1, slight; 2, moderate; 3, marked; 4, very marked. The level of erythema was scored using a scoring table with red taints. The cumulative score (erythema plus scaling plus thickening) served as a measure of the severity of inflammation (scale 0–12). At the days indicated, the ear thickness of the right ear was measured in duplicate using a micrometer (Mitutoyo).

### 2.6. MVD

Skin samples from the back lesions of mice were collected in 4% paraformaldehyde and embedded in paraffin. Sections (4 *μ*m) were added anti-Rabbit CD31 and goat anti-rabbit secondary antibodies and then observed the number of microvessels under a microscope (Olympus, Japan).

### 2.7. Cell Culture

The HUVEC and HaCaT cell lines were obtained from the Cell Culture Unit of Shanghai Science Academy (Shanghai, China). The cells were incubated with MEM (Gibco, USA)—containing 10% FBS (Hyclone, USA) at 37°C.

### 2.8. Western Blotting and Elisa

Skin samples were lysed, and the proteins were resolved in 10% SDS-PAGE. The membrane fraction was incubated with mouse-antitotal or phosphorylated Akt, GSK3-*β*, *β*-catenin, VEGF and Survivin antibodies, and rabbit-anti-*β*-actin antibody (Santa Cruz, CA), then IRDye 700DX- or 800DX-conjugated secondary antibodies (Rockland Inc., Gilbertsville, PA). Supernatant levels of total VEGF and Survivin were measured via enzyme-linked immunosorbent assay (ELISA) according to the manuals of VEGF and Survivin Elisa Assay kits (Nanjing Jiancheng Bioengineering Institute, Nanjing, China).

### 2.9. Immunochemical Staining and Immunofluorescence Staining

Skin samples from the dorsal lesions of mice were collected in 10% formalin and embedded in paraffin. Sections (5 *μ*m) were stained with hematoxylin and eosin (HE) and anti-Rabbit CD31, 4′,6-diamidino-2-phenylindole(DAPI), involucrin, and *β*-catenin, VEGF, and Survivin antibodies (Abcam, USA) diluted 1 : 100, and staining was assessed using light and fluorescence microscopes (Olympus, Japan).

### 2.10. Real-Time Polymerase Chain Reaction (RT-PCR)

Total RNA was extracted from skin lesions using TRIzol (Invitrogen, USA) and purified using a NucleoSpin RNA Clean-up Kit (Macherey-Nagel, Germany). Complementary DNA was generated using an Affinity Script Multiple Temperature cDNA Synthesis Kit (Agilent Technologies, USA) and specific primers ( Homo VEGF: forward: 5'-ATCCAATCGAGACCCTGGTG -3', reverse: 5'-ATCTCTCCTATGTGCTGGCC -3';Homo GAPDH: forward: 5‘- TCAAGAAGGTGGTGAAGCAGG -3', reverse: 5‘- TCAAAGGTGGAGGAGTGGGT -3'.), and the relative expression levels of genes were determined with an ABI 7500 Fast Real-Time PCR System using a real-time PCR master mix (Roche, USA). The actin gene was used as a reference to normalize the data.

### 2.11. Flow Cytometric Quantification of Apoptosis

HUVEC cells were harvested to assess apoptosis. After two washes with PBS at 1500 rpm for 5 min, the cells were resuspended in 500 *μ*L of Binding Buffer and mix 5 Annexin V-FITC (KeyGen Biotech, Nanjing) with 5 *μ*l PI (KeyGen Biotech, Nanjing) at room temperature for 5-15 min to light avoidance reaction (control group: normal cells without Annexin V-FITC and PI). Finally, the samples were analyzed using a FACSCalibur flow cytometer (Beckman, USA).

### 2.12. Cell Viability Assays

Cell viability was assessed using the Cell Counting Kit-8 (CCK8) assay (Beyotime C0037, USA). 10 *μ*L CCK8 was added to each well and incubated at 37°C for 4 h. The absorbance value of each well was detected by a microplate reader (Thermo Multiskan MK3, USA).

### 2.13. Statistical Analysis

The results were analyzed using SPSS for Windows version 21.0 (SPSS, Chicago, IL, USA) and expressed as mean ± standard deviation (SD). Between-group comparisons were performed using one-way ANOVA and analyzed by LSD *t*-test (when equal variances are assumed), and *P* < 0.05 indicated statistical significance.

## 3. Results

### 3.1. YXJD Fingerprint

**Figure 1:**
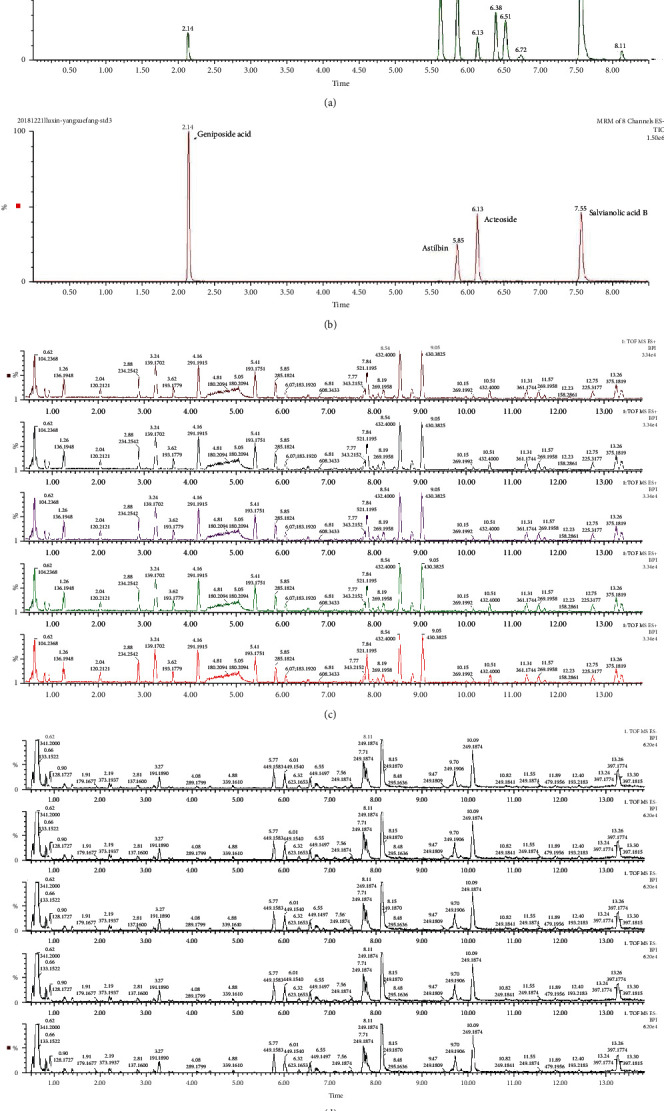
Total ion chromatograms of YXJD. Five groups of YXJD consisting of different batches were diluted to 0.06 g/mL and precipitated with an equal amount of methanol. The supernatants were obtained by centrifugation (10000 rpm) for 40 min and analyzed by UPLC-MS/MS using a mobile phase consisting of acetonitrile and 0.1% formic acid aqueous solution with a gradient program. The external standard one-point method was used to calculate the sample content based on the following formula: control sample injection quantity/sample peak area = sample injection quantity/sample peak area (a, b). Total ion flow in ESI positive mode. Counts (%) versus acquisition time (min) (c). Total ion flow in ESI anion mode. Counts (%) versus acquisition time (min) (d).

The components of 10 groups of YXJD consisting of different batches of herbs were analyzed by UPLC-MS/MS.  Figure 1(a) shows the total ion chromatograms of the 5 groups of standard samples; Figure 1(b) shows the total ion chromatograms of YXJD which displayed a high degree of similarity. As shown in Figure 1(c), 25 peaks in the total ion chromatograms of YXJD were assigned as common peaks, and the relative standard deviations of the relative retention time (RRT) of these 25 common peaks were lower than 1.0%, indicating that the RRTs of the 25 components are comparatively stable.

### 3.2. YXJD Ameliorated PASI Scores in a Mouse Model of PA

**Figure 2:**
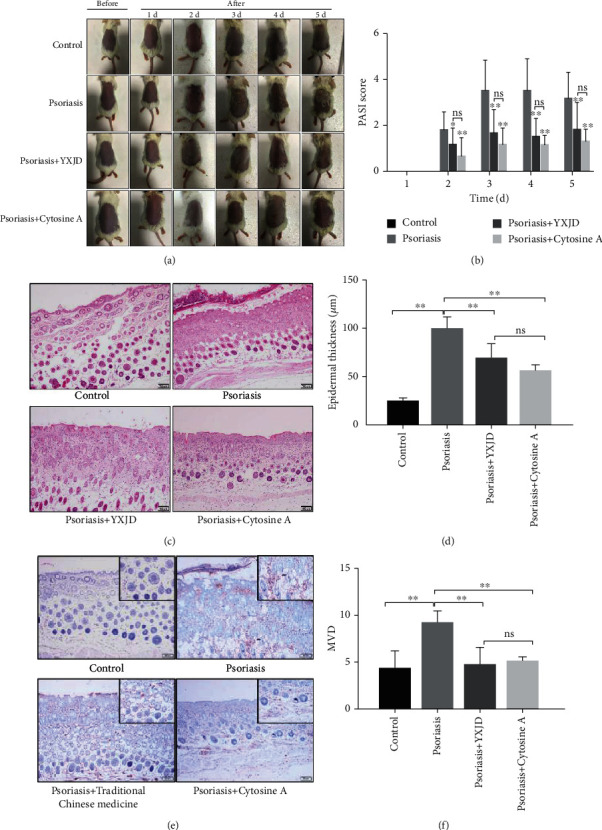
PASI scores, histomorphology, and MVD of the different groups. The effect of YXJD on IMQ-induced psoriasis-like lesions in mice (a). The PASI scores of the mice were evaluated from the 2^nd^ day to the 5^th^ day after model construction, and the PASI scores of each group were compared (b). The lesion samples were stained with hematoxylin and eosin (HE) (c), and the thickness was determined for each group (d). The microvessel density (MVD) of the four groups (e) was detected by immunocytochemistry. Bar = 100 *μ*m. ^∗^*P* < 0.05 vs. the model mice. The difference in MVD among the 4 groups showed that MVD in the group treated with YXJD was reduced (f).

On the 5^th^ day after the establishment of the psoriasis-like model, the psoriasis group exhibited inflamed thickened patches covered with silvery scales (Figure 2(a)). PASI scores were determined to evaluate the therapeutic effect of YXJD. The PASI score of the model group increased compared with the control group (Figure 2(b)) (*P* < 0.01). The PASI scores of the YXJD group and cyclosporin-A group were significantly lower than the score of the model group, demonstrating YXJD had a therapeutic effect on PASI scores in the PA mouse model, and its effect was equal to cyclosporin-A, which has a curative effect.

### 3.3. YXJD Improved Histomorphology in a Mouse Model of PA

HE staining was conducted to evaluate the effect of YXJD on histopathological changes in the lesion. The HE results (Figure 2(c)) show that the epidermal layer in the model group was obviously thicker and with incomplete keratinization. In the upper dermis, inflammatory cells infiltrated around capillaries, and the number of tortuous capillaries increased. The histological manifestations were psoriatic lesions in both the epidermis and dermis. However, YXJD and cyclosporin significantly reduced the thickness of the lesions in the IMQ-induced PA model.

### 3.4. YXJD Inhibited Microvessel Density (MVD) in a Mouse Model of PA

The number of microvessels was evaluated by measuring the microvessel density (MVD). The MVD in the lesions of the model group was increased compared with that in the control group, but the MVD was decreased distinctly in the groups treated with YXJD and cyclosporin. It showed that YXJD can reduce the number of microvessels, which might be a therapeutic mechanism for the treatment of psoriasis (Figure 2(e)).

### 3.5. YXJD Reduced the Levels of Survivin in a Mouse Model of PA

**Figure 3:**
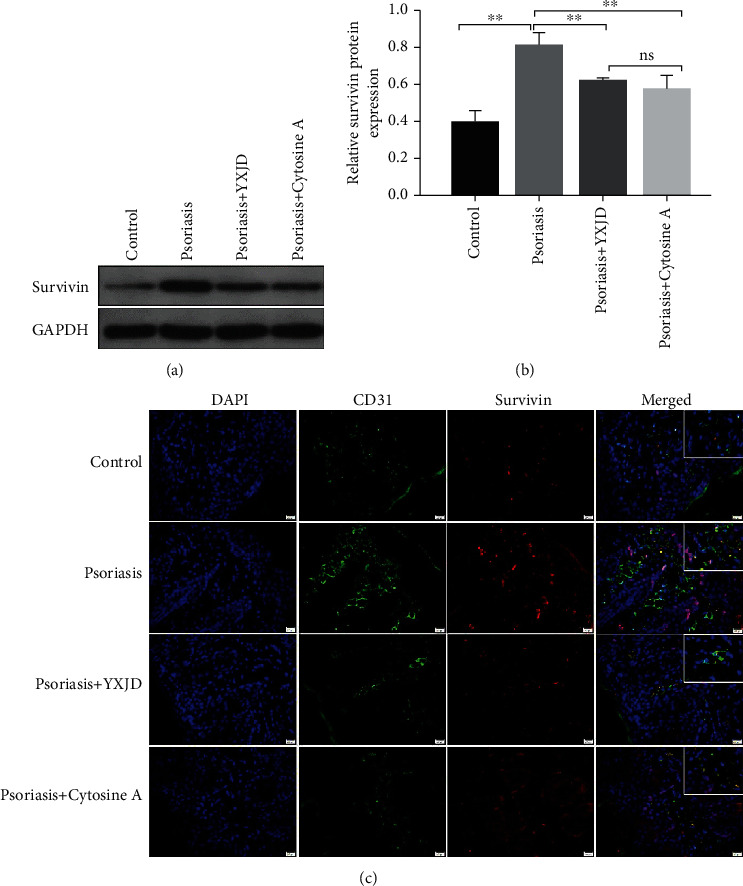
Protein levels and localization of Survivin, as detected by Western blot and immunochemical staining and immunofluorescence analysis. Survivin expression was evaluated by Western blotting (a). The expression of Survivin was increased in the model groups compared with the control groups, and YXJD inhibited the expression of Survivin in the lesions of IMQ-induced PA mice (b). Survivin protein expression in the mice was measured by immunochemical staining and immunofluorescence analysis (c). Double immunofluorescence staining for CD31 (green) and Survivin (red) in a representative skin sample from the 4 groups are shown. Nuclei were counterstained with DAPI (blue).

Survivin can affect the apoptosis of microvessels. The level of Survivin was increased in the psoriasis-like model mice (Figures 3(a) and 3(b)), and Survivin was mainly localized to the endothelial cells in the dermis (Figure 3(c)). YXJD inhibited the excessive expression of Survivin and had the same effect as that of cyclosporin.

### 3.6. The PI3K/Akt Pathway Mediated the Inhibitory Effects of YXJD in a Mouse Model of Psoriasis

**Figure 4:**
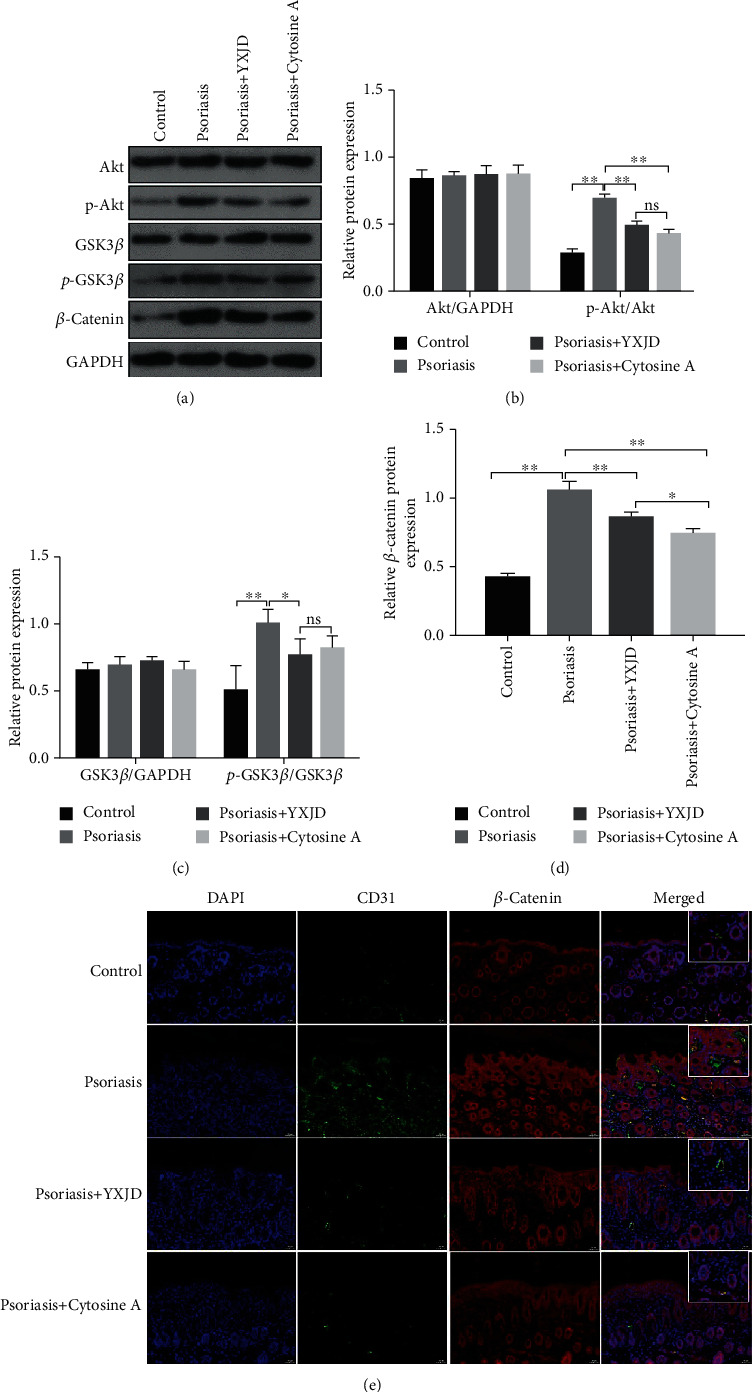
Protein levels of p-Akt, Akt, p-GSK3-*β*, GSK3-*β*, and *β*-catenin and the nuclear entry of *β*-catenin, as detected by Western blot, immunochemical staining, and immunofluorescence analysis YXJD inhibited the activation of the PI3K/Akt pathway. The levels of p-Akt, Akt, p-GSK3-*β*, GSK3-*β*, and *β*-catenin were reduced in YXJD-treated mice, as assessed by Western blotting (a–d). The nuclear entry of *β*-catenin (e) was decreased in the YXJD group mice compared with the model mice. Double immunofluorescence staining for CD31 (green) for vessels and *β*-catenin (red) in a representative skin sample from the 4 groups is shown. Nuclei were counterstained with DAPI (blue).

PI3K/Akt signaling interferes with important activities such as the apoptosis and proliferation of cells. Compared with those in the control group, the levels of Akt and GSK3-*β* phosphorylation and nonphosphorylated *β*-catenin were increased, as detected by Western blotting in the model group. The expression of the abovementioned proteins was decreased in the lesions of psoriasis-like mice treated with YXJD. YXJD and cyclosporin did not change the levels of Akt or GSK3-*β* phosphorylation, but cyclosporin had a stronger effect than YXJD in decreasing the expression of *β*-catenin (Figures 4(a)–4(d)). Immunochemical staining and immunofluorescence analysis of the nuclear entry of *β*-catenin in lesions showed that the number of endothelial cells in microvessels (CD31-labelled) increased in the lesions of IMQ-induced PA model mice; *β*-catenin was mainly observed in epidermal keratinocytes and endothelial cells in microvessels in the dermis (Figure 4(e)), but in YXJD-treated mice, the nuclear entry of *β*-catenin was reduced. These results suggest that YXJD might inhibit cell activity through a molecular mechanism involving the inhibition or interruption of the PI3K/Akt pathway to ameliorate the symptoms of PA model mice.

### 3.7. YXJD Reduced the Levels of VEGF in a Mouse Model of PA

**Figure 5:**
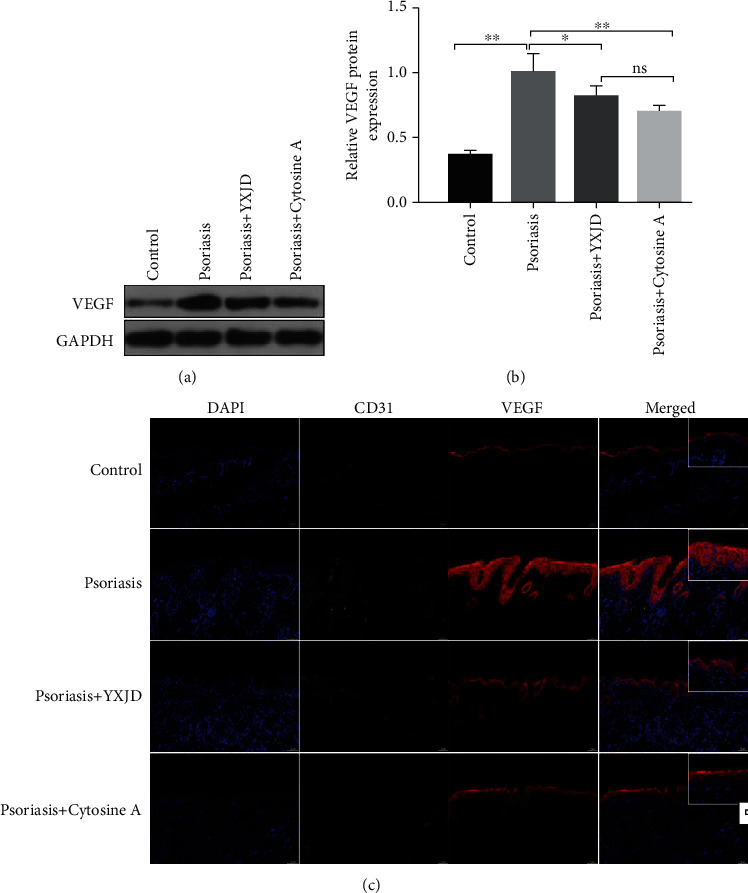
Protein levels and localization of VEGF, as detected by Western blot and immunochemical staining and immunofluorescence analysis VEGF expression, were evaluated by Western blotting (a). The expression of VEGF increased in the model groups compared with the control groups, and YXJD inhibited the expression of VEGF in the lesions of IMQ-induced PA mice (b). VEGF protein expression in the mice was measured by immunochemical staining and immunofluorescence analysis (d). Double immunofluorescence staining for CD31 (green), VEGF, and Survivin (red) in a representative skin sample from the 4 groups is shown. Nuclei were counterstained with DAPI (blue).

VEGF can affect the angiogenesis of microvessels. The expression of VEGF in the model group was increased significantly compared with that in the control group (Figures 5(a) and 5(b).), and we observed the expression of VEGF in keratinocyte and endothelial cells in the psoriasis-like model mice (Figure 5(c)). YXJD inhibited the excessive expression of VEGF in the corneum as well as corium of the lesion.

### 3.8. YXJD Ameliorates the Suppression of Autophagy in a Mouse Model of PA

**Figure 6:**
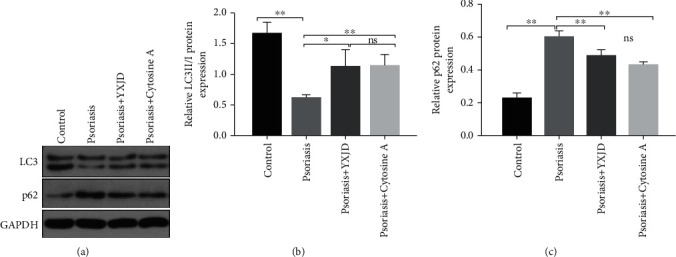
Protein levels of LC3II/I and p62, as detected by Western blot. LC3II/I and p62 expression were evaluated by Western blotting (a–c).

Autophagy-associated proteins, LC3II, LC3I, and p62 proteins, can show the occurrence of autophagy. The expression of LC3II/I protein in the model group was decreased compared with that in the control group (Figures 6(a) and 6(b)), while the expression of p62 protein, on the contrary, was significantly increased in the model group compared with the control group (*P* < 0.05) (Figures 6(a) and 6(c)). The downregulation of LC3II/I protein and upregulation of p62 protein demonstrate that the autophagy in psoriatic mice is significantly decreased. While the level of LC3II/I protein increased and p62 protein decreased in mouse model treated by YXJD and YXJD inhibited the excessive suppression of autophagy in PA lesions and had the same effect as that of cyclosporin, which suggests a decreased autophagic potential for YXJD.

### 3.9. The Effect of YXJD on HUVEC Cells Induced by Survivin and Its Molecular Mechanism

**Figure 7:**
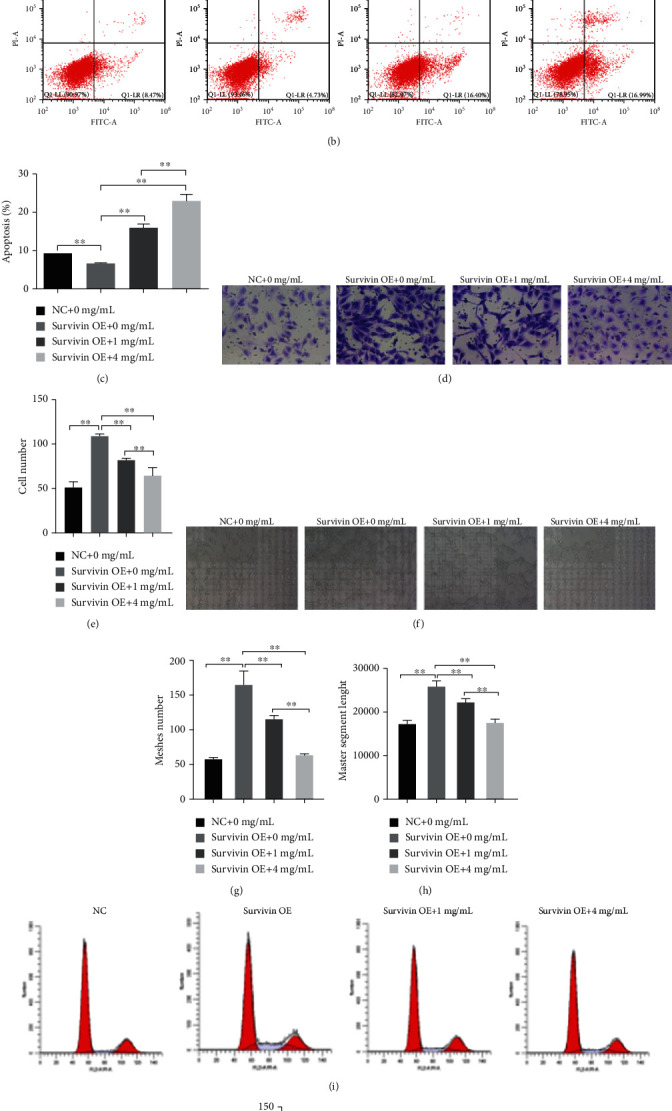
The effect of YXJD on HUVEC cells induced by Survivin and its molecular mechanism HUVEC cells transfected with Survivin overexpressed plasmid were treated with different concentrations of YXJD (0, 1, and 4 mg/mL) for 24 h by CKK-8 detection (a). The induction of apoptosis was determined by annexin V–FITC/PI staining assay (b, c). Transwell: the effect of different concentrations of YXJD (0, 1, and 4 mg/mL) on the migration ability of HUVEC cells transfected with Survivin overexpression plasmid (d). Statistical chart: ordinate: number of cells (e). Tube formation detection was used to detect the effects of different concentrations of YXJD on the angiogenic ability of HUVEC cells transfected with Survivin overexpression plasmid (f). Meshes number (g) and master segments length (h). Statistical chart: ordinate: number of lumen/length of lumen. Magnification: 100× (f–h). Flow cytometry cell detected cycle distribution of HUVEC cells transfected with Survivin overexpression plasmid with different concentrations of YXJD (i). Statistical chart: ordinate: cell number (j).

Survivin overexpression can significantly increase cell viability, and different concentrations of YXJD can inhibit HUVEC cell viability, which is dose-dependent. After the preliminary experiment, we screened out the appropriate concentration of YXJD (1 mg/mL and 4 mg/mL) (the supplementary materials for details). In HUVEC cells transfected by Survivin overexpression plasmid (Survivin OE), we observed that the cell viability increased compared with the HUVEC cells transfected by normal control plasmid (NC), and when different concentrations of YXJD (0, 1, and 4 mg/mL) intervened into Survivin OE, we found YXJD could inhabit the cell viability (Figure 7(a)). The apoptosis level of Survivin OE with 4 mg/mL YXJD treatment group was significantly higher than Survivin OE with 1 mg/mL treatment group (Figures 7(b) and 7(c)).

Compared with the NC group, the migration ability of HUVEC cells rose after Survivin overexpression plasmid transfection, and YXJD could inhibit the increase of HUVEC cell migration caused by Survivin overexpression, and the inhibitory effect of a high concentration of YXJD on HUVEC cells migration was prominent (*P* < 0.01) (Figures 7(d) and 7(e)). In the Survivin OE group, the ability of HUVEC cells was increased as well as the meshes number and length of the master segment length (Figures 7(f)–7(h)). The tube formation ability of HUVEC cells decreased, and the lumen length became shorter induced by high concentrations of YXJD. Therefore, transfection of Survivin overexpression plasmid can increase the tube-forming ability of HUVEC cells, while YXJD inhibited the tube formation ability of HUVEC cells to a certain extent, and the inhibition of HUVEC cells migration induced by a high concentration of YXJD was enhanced. After Survivin overexpression plasmid transfection, the proportion of Survivin OE group in G0/G1 phase was evidently lower than that in the NC group, while that in S phase was opposite. The proportion of Survivin OE group treated with different concentrations of YXJD in G0/G1 phase was relatively increased and in S phase was decreased (Figures 7(i)–7(j)).

### 3.10. YXJD Inhibited the Expression of Survivin and the Decrease of Autophagy, PI3K/Akt Pathway Relative Proteins, and VEGF in HUVEC Cell Transfected Survivin Overexpression Plasmid

**Figure 8:**
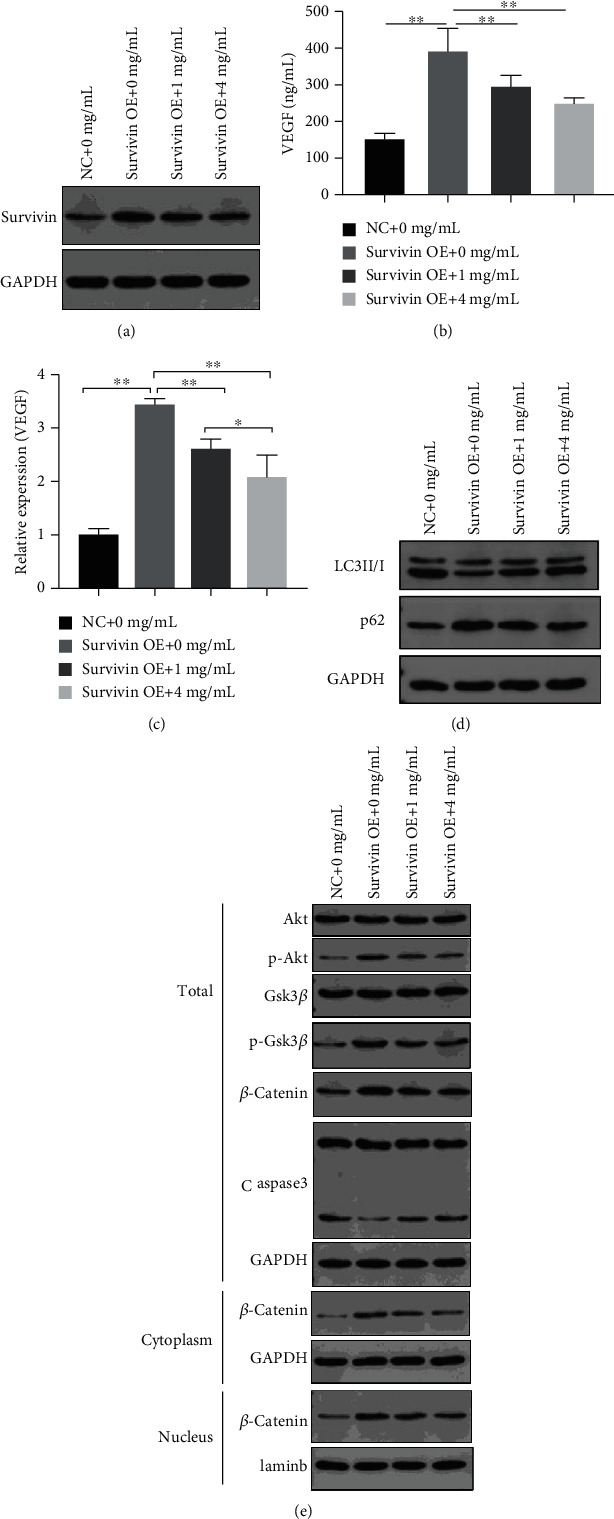
Western blot: the effect of different concentrations of YXJD on the expression of Survivin (a), LC3II/I, and p62 (d) in HUVEC cells transfected with Survivin overexpression plasmid, and the expression of PI3K/Akt- *β* catenin pathway and Caspase3 (e). ELISA: the effect of different concentrations of YXJD (0, 1, and 4 mg/mL, for 72 h) on the secretion of VEGF in HUVEC cells transfected by Survivin overexpression plasmid (b). qRT-PCR: the effect of different concentrations of YXJD on the synthesis of VEGF in HUVEC cells transfected by Survivin overexpression plasmid (c). GAPDH was used as the internal reference for cytoplasmic protein content and total protein content; Laminb as the internal reference for nuclear protein content; total stands for total protein; cytoplasm for cytoplasmic protein; nucleus for nuclear protein.

To observe the effect of YXJD on the expression of Survivin and the decrease of autophagy, PI3K/Akt pathway relative proteins, and VEGF, we detected the proteins by ELISA, Western blot, and qRT-PCR. The result indicated that the expression of Survivin, p62, PI3K/Akt pathway relative proteins, and VEGF relative expressions increased and LC3II/I decreased in the Survivin OE group. YXJD can inhibit the expression of Survivin and VEGF in a dose-dependent manner (Figures 8(a)–8(c)). For the expression of PI3K/Akt pathway relative proteins, the result showed that the expression of Akt and GSK3*β* protein in each group has no significant change in all groups, while in the Survivin OE group, the total protein content of p-Akt, p-GSK3*β*, *β*-catenin, and the entry of *β*-catenin into the nucleus promoted, on the contrary, Caspase3 shearing decreased. After being treated by YXJD, the phosphorylation level of Akt and GSK-3*β* and the content of *β*-catenin decreased and the shearing of Caspase3 increased, all of the groups in a dose-dependent manner (Figure 8(e)). The expression of LC3II/I protein decreased, while the level of p62 increased in the Survivin OE group. YXJD could increase the content of LC3II/I and reduce the content of p62, which demonstrated YXJD could inhibit the downregulation of autophagy in HUVEC cells caused by the overexpression of Survivin (Figure 8(d)).

## 4. Discussion

Survivin is a member of the inhibitor family of apoptosis protein discovered by Ambrosini et al. in 1997 [15] and is currently the strongest protein that inhibits apoptosis. It participates in the inhibition of apoptosis by inhibiting the signaling pathway and regulating the apoptosis protein of the Caspase family, while interfering different cell cycles to participate in the mitotic activity of cells. The apoptosis of microvessels plays an important role in the occurrence and development of psoriasis. Abnormal apoptosis leads to a relative increase in the number of microvessels. In the terminal stage of psoriasis, stilled microvessels are related to the apoptosis inhibition of vascular endothelial cells. The process of apoptosis in psoriasis is regulated by several factors, such as the Bcl-2 family of proteins, the inhibitor of apoptosis (IAP) family of proteins, and the caspase family of proteins. Koch et al. [16] first discovered that the expression of Survivin is significantly increased in the lesions of psoriasis. Survivin can participate in the processes of angiogenesis and apoptosis. When Survivin acts on subdermal microvascular endothelial cells, normal physiological apoptotic activity in microvessels in the downstream apoptotic pathway can be suppressed by regulating the expression of apoptotic proteins such as caspase-3 and Bcl-2, and the relative number of microvessels increases, which is an important pathological cause of psoriasis, leading to erythema. Survivin mRNA and protein expression were significantly higher in lesions than in nonlesional skin in psoriasis patients [17]. Survivin mRNA expression was positive in the peripheral blood of patients with psoriasis but not in normal patients [18]. In the psoriasis-like mice, we observed increased Survivin staining for CD31 in representative psoriasis-like lesions, which suggests that YXJD can inhibit angiogenesis under pathological conditions. In vitro, we used HUVEC cells transfected with Survivin overexpressed plasmid as psoriasis cell model and found YXJD reduced the activity of HUVEC cells in a dose-dependent manner as well as the secretion of VEGF. Therefore, YXJD ameliorates the overexpression of Survivin in psoriasis.

Autophagy plays a crucial role in cell growth development and occurrence of diseases, which is a highly conservative protein degradation pathway from eukaryotic cells to humans and is essential for removing protein amounts and misfolded proteins in healthy cells. More and more evidences show that autophagy plays a vital role in cell survival, aging, and homeostasis, which is associated with many diseases, including psoriasis, cancer, inflammatory disease, infectious diseases, and metabolic diseases [19–22]. Immune functions, such as intracellular bacterial clearance, inflammatory factor secretion, and lymphocyte development, are impacted by autophagy-related proteins. Autophagy runs through the pathogenesis of psoriasis and is an important target for the treatment of psoriasis. Autophagy runs through the pathogenesis of psoriasis and is an important target for the treatment of psoriasis. The infiltration of T lymphocytes is an important pathogenesis that mediates psoriasis. IL-17 produced by T-helper (Th) 17 cells plays a key role in the occurrence of autoimmunity and allergies [23], and its level is elevated in psoriasis [24]. mTOR also plays a critical role in regulating autophagy [25]. IL-17A stimulates keratinocytes to activate the PI3K/AKT/mTOR signal and inhibits autophagy by suppressing the formation of autophagosomes simultaneously [26]. Excessive proliferation of keratinocytes is another important pathological manifestation of psoriasis [27], and it is also the key to the treatment of psoriasis. In human keratinocyte, the expression of p62 which is the autophagy negatively regulated protein is essential to prevent excessive inflammation and induce cathelicidin. TLR2/6 or TLR4 in keratinocytes activates the expression of p62 by inducing NADPH oxidase 2 and 4 and generating reactive oxygen species [28]. We found autophagy is repressed in psoriasis-like mice, which is in line with Pallavi's research. At present, there are few studies on autophagy of microvessels in psoriasis. Our results show that autophagy is inhibited in HUVEC cells transfected by Survivin overexpression plasmid, and YXJD can improve the disorder. However, the occurrence of autophagy is intricate that various factors can affect it. In the future, we will do more in-depth studies on how YXJD can interfere with autophagy in microvessels of psoriasis.

The relationship between autophagy and apoptosis is intricate. Bcl family proteins inhibit the occurrence of autophagy after binding to Beclin1 [29]. In reverse, the occurrence of autophagy also inhibits apoptosis. After the inoculation of Peste des petits ruminants virus (PPRV) in goat endometrial epithelial cells (EECS), cell autophagy is activated and apoptosis is suppressed, and after knocking out autophagy-related genes Beclin-1 and ATG7, apoptosis occurred [30]. The research [31] observing repressive LC3 and Beclin1 levels in the lesions of imiquimod-induced and IL-33 intraperitoneal injection psoriasis model mice that demonstrated the autophagy and apoptosis is suppressed, which, the results of autophagy, is consistent with our experimental results. The relationship between autophagy and apoptosis is complex and in most diseases, autophagy, and apoptosis trade-off. In our study, we found that the expression of the inhibitory apoptosis protein Survivin rose in psoriatic mouse model lesions, which indicated that apoptosis was suppressed; while the expression of LC3II/I was decreased and p62 protein was active in psoriatic mouse model lesions and Survivin overexpressing HUVEC cells, which manifested the autophagy was inhibited. Our experimental results observed that both autophagy and apoptosis in the lesions of psoriasis model mice reduced, which is pivotal in vascular regression in psoriasis. Besides, YXJDD can alleviate the suppression of autophagy in psoriasis lesions. Studies on the relationship between apoptosis and autophagy in psoriasis remain limited, which desiderates more research to verify.

**Figure 9:**
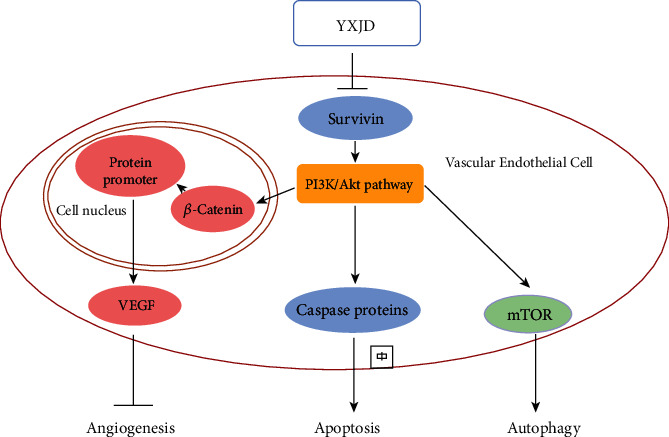
YXJD regulated vascular regression via Survivin/PI3K/Akt pathway in morbigenous signal propagation.

The PI3K/Akt pathway interferes with cell apoptosis, proliferation, and other important activities. The PI3K family is related to the regulation of cell activities. Akt plays an important role in maintaining the physiological function of cells such as apoptosis, proliferation, membrane transportation, and secretion. Increasing the expression of p-GSK3-*β* results in the inactivation of GSK3-*β*, which leads to the accumulation and activation of *β*-catenin [32]. *β*-Catenin enters the nucleus to promote the DNA transcription of proteins important for cell proliferation and migration, such as Survivin. *β*-catenin can not only regulate the production of VEGFR-2 but can also regulate the level of VEGF and relative mRNA expression [33, 34]. We found that the level of VEGF and the PI3K/AKT pathway-related proteins activated after HUVEC cells transfected with Survivin overexpression plasmid, The results demonstrate Survivin overexpression can stimulate the secretion of VEGF via PI3K/AKT pathway. PI3K activates AKT and then activates tuberous sclerosis complex 1/2(TSC1/TSC2) and phosphorylates TSC2, which suppressed the TSC1/TSC2 complex and enables mTOR activation [35–37]. In psoriasis lesions of IQM-induced mice and HUVEC cells transfected with Survivin overexpression plasmid, we observed the activation of PI3K/AKT pathway, and YXJD could alleviate such morbigenous signal propagation (Figure 9).

In our study, Survivin regulated the VEGF via PI3K/Akt pathway, which exacerbated vascular regression in psoriasis. VEGF is a proangiogenic factor that plays an important role in angiogenesis [38]. The earliest pathological process of psoriasis is the angiogenesis of microvessels in the dermal papilla [9]. Angiogenesis occurs throughout psoriasis. In the early period of psoriasis, the capillaries of the dermal papilla exhibit abnormal expansion. Excessive dilatation and increased permeability of the venous branches of the capillary plexuses of the dermal papilla result in a large amount of inflammation and chemokine exudation. The process of angiogenesis in psoriasis is regulated by several factors, such as VEGF, MMPs, and TGF-*β*. VEGF is the most important mediator of angiogenesis under physiological and pathological conditions. It can increase microvessel permeability, promote endothelial cell division and proliferation to lead to angiogenesis, and has a tendency towards inflammatory cells and endothelial cells, which are the main causes of vascular changes. VEGF is synthesized in keratinocytes and endothelial cells in the skin. Clinical studies have confirmed that the level of VEGF in the skin lesions of patients with psoriasis is significantly higher than that in the nonlesion areas and normal skin, and that the level of VEGF is related to the severity of the disease [39] and is also significantly increased in the serum [40]. In our study, we found that the level of VEGF was increased, and YXJD inhibited the excessive expression of VEGF as well as the secretion of VEGF and related proteins expression in HUVEC cells transfected by Survivin overexpression and IMQ-induced psoriasis-like mouse model, which inferred YXJD can ameliorate the abnormal neovascularization in psoriasis (Figure 9.).

## 5. Conclusion

In conclusion, we demonstrate YXJD alleviates IMQ-induced psoriasis-like pathological changes in microvessels by inhibiting the expression of Survivin in endothelial cells via Survivin/PI3K/Akt pathway, which are related to apoptosis, autophagy, and angiogenesis of microvessels in psoriasis. Our study provides good evidence for the targeted treatment of psoriasis with TCM, and we expect to study more on the effect of effective component in YXJD in the future.

## Data Availability

The data used to support the findings of this study are available from the corresponding author upon request.

## Supplementary Materials

Supplementary Materials Figure 1: running gum chart: PCR product recovery gel (a). Double enzyme digestion to recover the running rubber (b). PCR identification running map (c). Sequencing analysis (d). Western Blot: the effect of Survivin overexpression plasmid transfection on the expression of Survivin in HUVEC cell (e). Figure 2: CCK-8 was used to detect the effect of different concentrations of YXJD on HUVEC cell activity. Data statistics were analyzed by two-way ANOVA analysis of variance. Error bars indicate SD, ^∗∗^ indicates *P* < 0.01 vs. 0 mg/mL group at the same test time. Figure 3: the effect of different concentrations of YXJDD (0, 0.01, 0.05, 0.2, 1, 5, and 20 mg/mL) on cell viability of HUVEC cells transfected with control plasmid (a) and Survivin overexpression plasmid (b) for 24 h, 48 h, and 72 h by CCK8. Figure 4: HUVEC cells transfected with Survivin overexpressed plasmid were treated with 0.25, 0.5, or 1 mg/ml YXJDD for 24 h. The induction of apoptosis was determined by annexin V–FITC/PI staining assay (a). The apoptosis rate of HUVEC cells (b). The same drug concentration at the same time.
