# Supplementary material for: Yangxue Jiedu Fang Ameliorates Psoriasis by Regulating Vascular Regression via Survivin/PI3K/Akt Pathway
Source: J Immunol Res. 2021 Jan 8;2021:4678087. doi: 10.1155/2021/4678087 (PMC7834796; doi:10.1155/2021/4678087)
Supplement: Supplementary Materials — Figure 1: running gum chart: PCR product recovery gel (a). Double enzyme digestion to recover the running rubber (b). PCR identification running map (c). Sequencing analysis (d). Western Blot: the effect of Survivin overexpression plasmid transfection on the expression of Survivin in HUVEC cell (e). Figure 2: CCK-8 was used to detect the effect of different concentrations of YXJD on HUVEC cell activity. Data statistics were analyzed by two-way ANOVA analysis of variance. Error bars indicate SD, ∗∗ indicates P < 0.01 vs. 0 mg/mL group at the same test time. Figure 3: the effect of different concentrations of YXJDD (0, 0.01, 0.05, 0.2, 1, 5, and 20 mg/mL) on cell viability of HUVEC cells transfected with control plasmid (a) and Survivin overexpression plasmid (b) for 24 h, 48 h, and 72 h by CCK8. Figure 4: HUVEC cells transfected with Survivin overexpressed plasmid were treated with 0.25, 0.5, or 1 mg/ml YXJDD for 24 h. The induction of apoptosis was determined by annexin V–FITC/PI staining assay (a). The apoptosis rate of HUVEC cells (b). The same drug concentration at the same time. [file 4678087.f1.docx]

# Supplementary Materials

**1.** **Survivin overexpression plasmids construction**

Before we observed the effect of YXJD on HUVEC cells induced by Survivin and its molecular mechanism, we constracted the Survivin overexpression plasmids. The PCR product recovery gel size is 400bp, which is in line with Survivin size 429bp (Fig.1. (a)). A double-digested band is around 400 and one is around 4500, which is in accordance with Survivin size 429bp and vector size 4472bp (Fig.1. (b)). PCR identification of the four colonies all met the requirements (Fig.1. (c)). The sequencing results show that the 100% comparison is successful (Fig.1. (d)), indicating that the vector was successfully constructed. The successfully constructed vector was transfected into HUVEC cells, and 72 hours later, WB detection was performed. The results showed that cells transfected with Survivin overexpression vector can express Survivin highly (Fig.1. (e)), indicating that the vector has good effect and subsequent experiments can be performed.


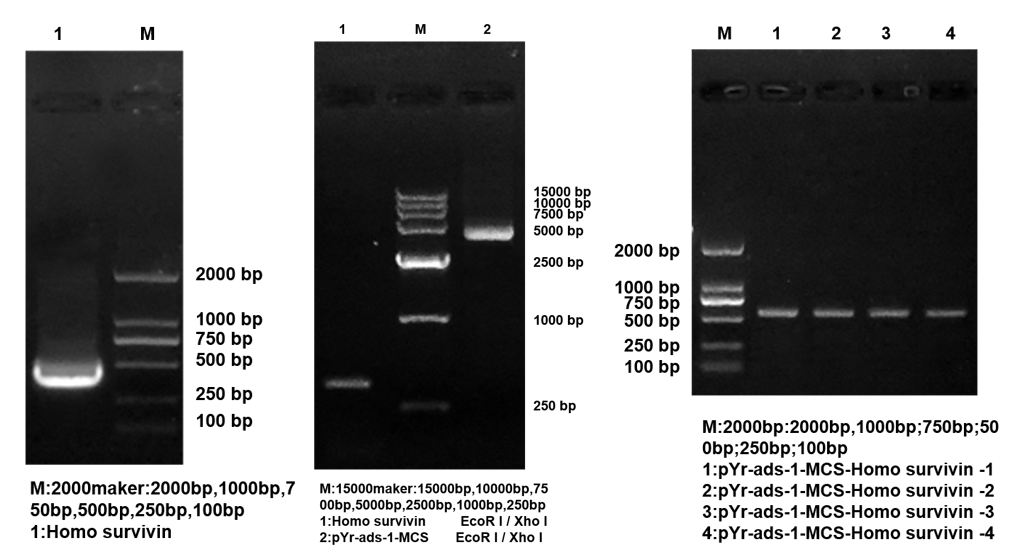


**(a) (b) (c)**


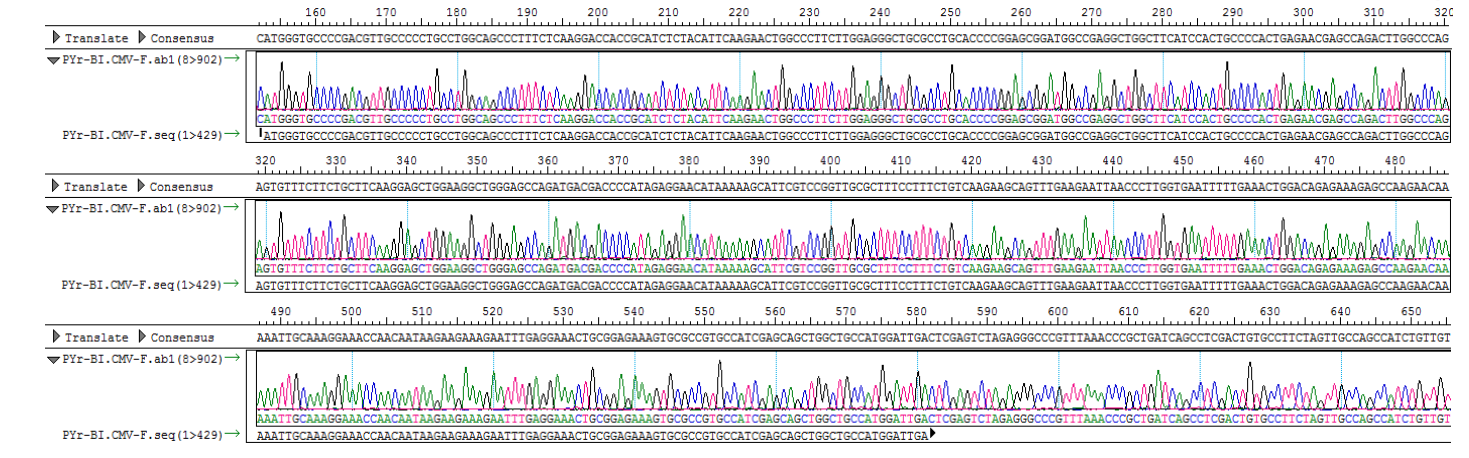


**(d)**


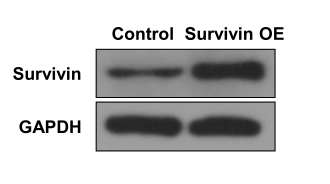


(e)

***Fig.1.*** Running gum chart: PCR product recovery gel(a). Double enzyme digestion to recover the running rubber(b). PCR identification running map(c). [Sequencing](file:///C:\Users\aa\AppData\Local\youdao\dict\Application\7.5.0.0\resultui\dict\?keyword=sequencing)[analysis(d).](file:///C:\Users\aa\AppData\Local\youdao\dict\Application\7.5.0.0\resultui\dict\?keyword=analysis) Western Blot: the effect of Survivin overexpression plasmid transfection on the expression of Survivin in HUVEC cell(e).

**2. YXJD initial screening of concentration range by CCK-8 detection**

Before we observed the effect of YXJD on HUVEC cells, we screened for different concentrations of YXJD. The constructed vector was transfected into HUVEC cells and 72 hours late it showed the cells transfected with Survivin overexpression vector could highly express Survivin. Control denotes HUVEC cells transfected with control plasmid, Survivin OE represents HUVEC cells transfected with Survivin overexpression plasmid. The effect of different concentrations of YXJDD (0,0.01,0.05,0.2,1,5,20 mg/mL) on the cell viability of HUVEC cells for 24 h and 48 h by CCK8.


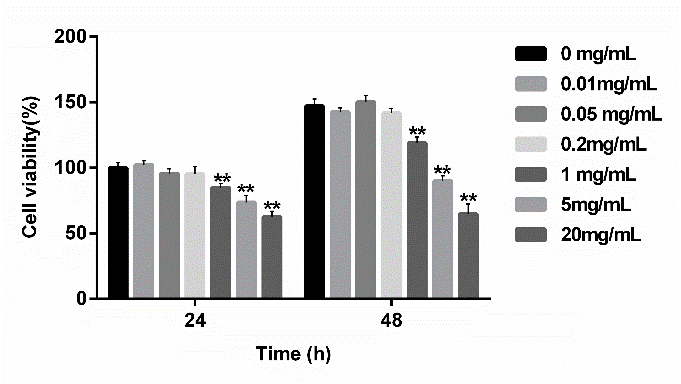


***Fig.2.*** CCK-8 was used to detect the effect of different concentrations of YXJD on HUVEC cell activity. Data statistics were analyzed by Two-WayANOVA analysis of variance. Error bars indicate SD, ** indicates p <0.01 vs. 0mg / mL group at the same test time.

**3.** **The effect of YXJD on cytotoxicity of control and the activity and apoptosis of survivin overexpressing cells**

**3.1 Cell viability by CCK-8 detection**

HUVEC cells were transfected with control plasmids or survivin overexpression plasmids for 72 hours, and then treated with different YXJD concentrations (0, 0.0625, 0.125, 0.25, 0.5, 1 mg /ml) for 0, 24, 48, and 72 hours and then detected by CCK8 for cell viability. The result showed 0.5mg/ml YXJD at 24h and 0.25mg/ml YXJD at 48h are not toxic to normal cells but inhibits the activity of survivin overexpressing cells, and the inhibitory effect is minute.


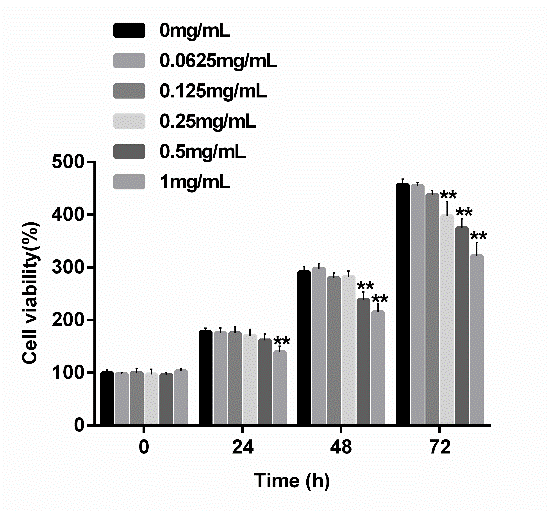

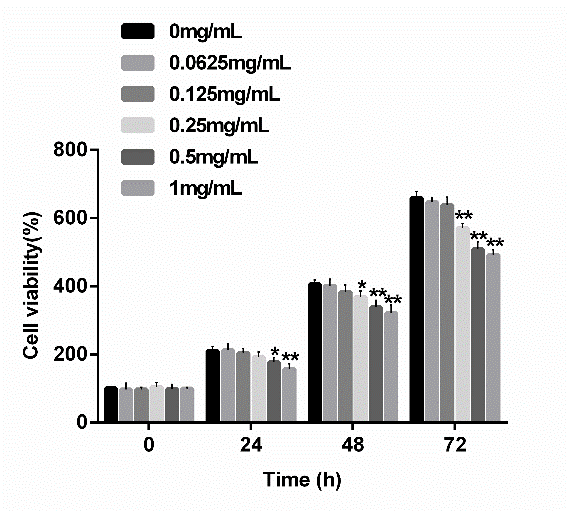


(a) (b)

***Fig.3.*** The effect of different concentrations of YXJDD (0, 0.01, 0.05, 0.2, 1, 5, 20 mg/mL) on cell viability of HUVEC cells transfected with control plasmid(a) and Survivin overexpression plasmid(b) for 24 h, 48 h, 72h by CCK8.

**3.2 Apoptosis detection by fow cytometry detection**

To investigate the effect of YXJD on cytotoxicity of control cells as well as the viability and apoptosis in Survivin overexpression HUVEC cells, we treated HUVEC cells transfected with control plasmid or survivin overexpression plasmid for 72 h with different concentrations of YXJD. The induction of apoptosis was determined by annexin V–FITC/PI staining assay(Fig.4. (c-d)). At the same time, compared with the cells without drug treatment, the apoptosis rate of cells treated with different concentrations of YXJD increased (the apoptosis rate of control plasmid transfected cells had no significant change after 0.25 mg/mL drug treatment at 48h) in a dose-dependent manner. And the rangeability of apoptosis rate in control group was significantly greater than that in Survivin overexpression plasmid group. It is suggested YXJDD can induce apoptosis in the group of Survivin overexpression plasmid but it is also toxic to the control group.

Survivin overexpression can significantly increase cell activity and different concentrations of YXJDD can inhibit cell activity which is dose-dependent. The inhibition rates of 0.25-32mg/mL respectively were 12.67%, 13.22%, 24.63%, 33.80%, 53.81%, 60.72%, 74.20% and 85.49%. We selected 1mg/ml and 4mg/ml for follow-up experiments.


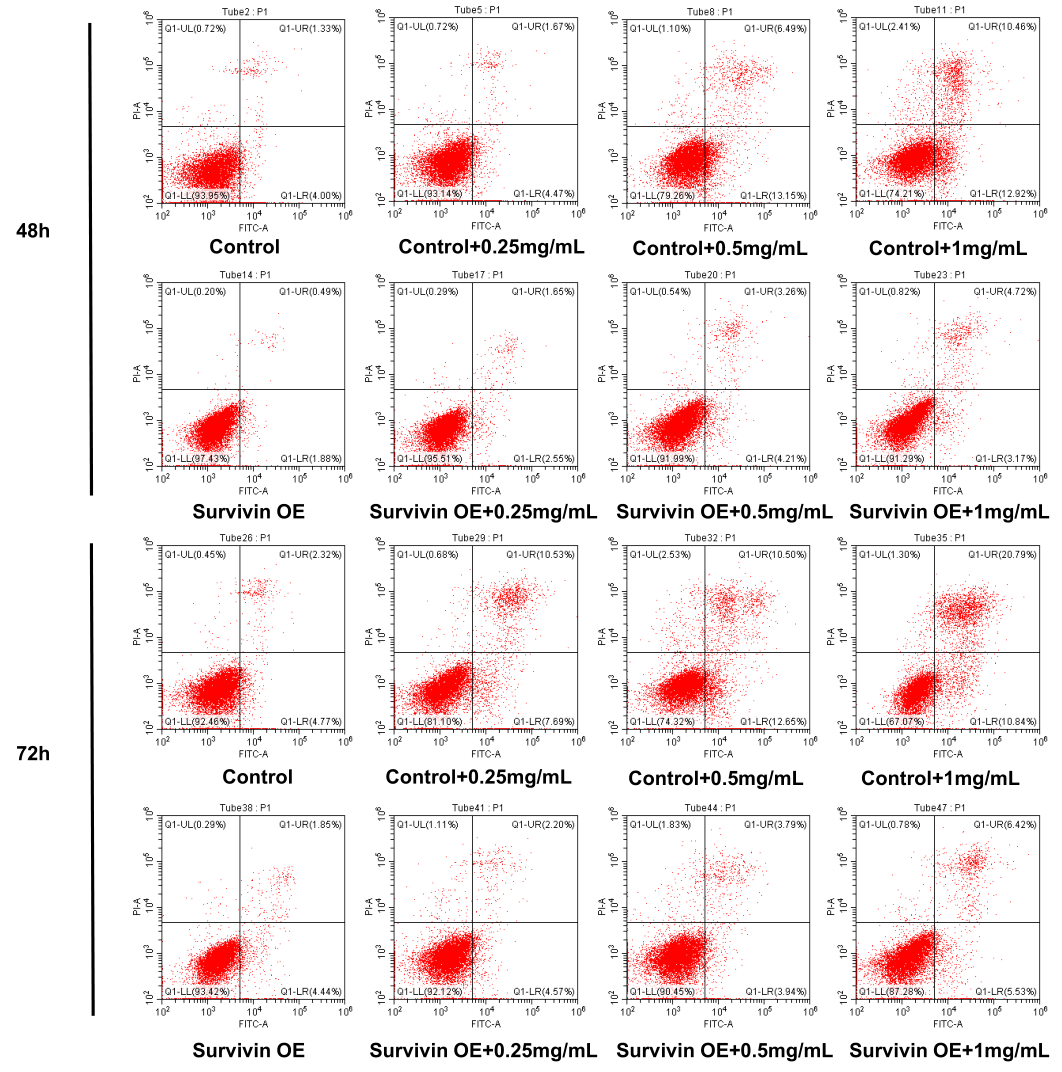


(a)


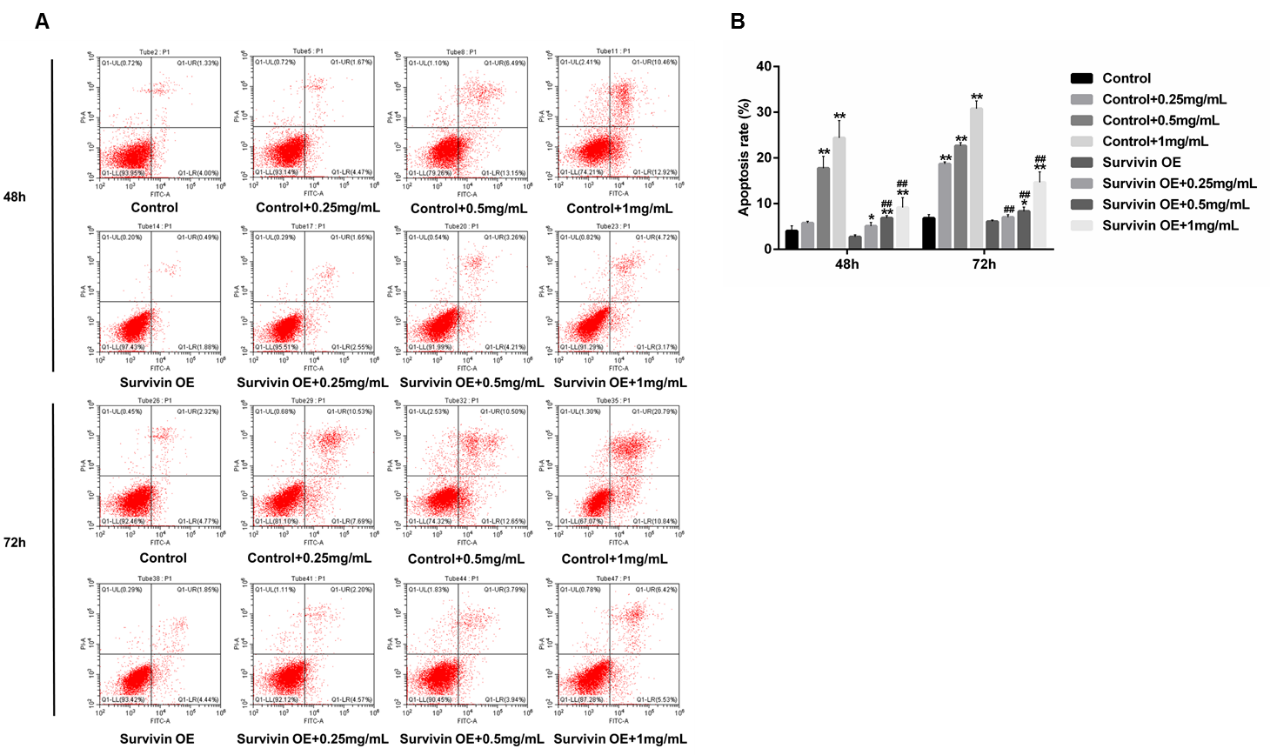


(b)

***Fig.4.*** HUVEC cells transfected with Survivin overexpressed plasmid were treated with 0.25, 0.5 or 1 mg/ml YXJDD for 24 h. The induction of apoptosis was determined by annexin V–FITC/PI staining assay(a). The apoptosis rate of HUVEC cells(b). The same drug concentration at the same time.
